# Implementing a Farmers’ Market Incentive Program: Perspectives on the New York City Health Bucks Program

**DOI:** 10.5888/pcd10.120285

**Published:** 2013-08-29

**Authors:** Gayle Holmes Payne, Holly Wethington, Lauren Olsho, Jan Jernigan, Rosanne Farris, Deborah Klein Walker

**Affiliations:** Author Affiliations: Holly Wethington, Jan Jernigan, Rosanne Farris, National Center for Chronic Disease Prevention and Health Promotion; Centers for Disease Control and Prevention, Atlanta, Georgia; Lauren Olsho, Deborah Klein Walker, US Health Division, Abt Associates Inc, Cambridge, Massachusetts.

## Abstract

**Introduction:**

One strategy for lowering the prevalence of obesity is to increase access to and affordability of fruits and vegetables through farmers’ markets. However, little has been documented in the literature on the implementation of such efforts. To address this gap, the Division of Nutrition, Physical Activity, and Obesity (DNPAO) sponsored an evaluation of the New York City Health Bucks program, a farmers’ market coupon incentive program intended to increase access to fresh fruits and vegetables in underserved neighborhoods while supporting local farmers.

**Methods:**

We conducted a process evaluation of Health Bucks program implementation. We interviewed 6 farmer/vendors, 3 market managers, and 4 program administrators, and collected data on site at 86 farmers’ markets, including surveys of 81 managers and 141 farmer/vendors on their perspectives on promotion and redemption of the incentive coupons; knowledge and attitudes regarding the program; experiences with markets and products; and facilitators and barriers to program participation.

**Results:**

Results indicate that respondents view Health Bucks as a positive program model. Farmers’ market incentive coupon programs like Health Bucks are one strategy to address the problem of obesity and were associated with higher fruit and vegetable access and purchases in low-income communities.

**Conclusions:**

This evaluation identified some areas for improving implementation of the Health Bucks program. Farmers’ market incentive programs like Health Bucks may be one avenue to increase access to and affordability of fruits and vegetables among low-income persons. Further research is needed to assess the potential effects of these programs on access and health outcomes.

## Introduction

US obesity prevalence in the past 30 years has more than doubled among adults and more than tripled among youth (1). One contributor to obesity is poor-quality diets, including inadequate fruit and vegetable consumption (2). Although scant evidence directly links fruit and vegetable consumption to obesity prevention, indirect evidence indicates that eating fruits and vegetables may facilitate weight reduction or maintenance (3–6). The Division of Nutrition, Physical Activity, and Obesity (DNPAO) at the Centers for Disease Control and Prevention (CDC) provides guidance to increase access to and availability and affordability of fruits and vegetables as one obesity-prevention strategy. One initiative that demonstrates this guidance is farmers’ markets.

Farmers’ markets are recurring gatherings of farmers selling their food products, including fruits and vegetables, directly to consumers. The number of farmers’ markets in operation has more than quadrupled since 1994, from 1,755 to 7,175 (7). Farmers’ market access may be associated with increased fruit and vegetable consumption (8–10). Additionally, farmers’ markets are good sources for locally grown fresh fruits and vegetables in urban and low-income settings (11–13).

Low-income families and participants in the US Department of Agriculture’s (USDA’s) nutrition programs such as the Supplemental Nutrition Assistance Program (SNAP) and the Special Supplemental Nutrition Program for Women, Infants, and Children (WIC) consume fewer fruits and vegetables than recommended by current guidelines (14,15). As a result, to allow low-income families to overcome barriers of cost and availability, some nutrition programs have extended benefits to farmers’ market purchases for fruits and vegetables via coupons or electronic benefit transfer (EBT) debit cards (16). The USDA manages the EBT system by allowing recipients to authorize transfer of their government benefits from a federal account as payments to retailers. EBT has been implemented in all states since June of 2004. In recent years, wireless EBT technology has increasingly been adapted for use in farmers’ markets.

Although farmers’ market incentive programs can increase access to high-quality produce, there is a dearth of literature on implementation of such efforts. Other evaluations have focused on specific components of farmers’ markets (eg, food hygiene [hygiene standards and food safety]) (17) and consumer interaction (18). To help address this gap, in 2011 DNPAO contracted with Abt Associates, Inc. to conduct an evaluation of the New York City (NYC) Health Bucks program.

Health Bucks is a pioneering farmers’ market incentive program intended to reduce food insecurity among NYC residents by increasing access to and affordability of fresh fruits and vegetables through farmers’ markets in low-income neighborhoods. Health Bucks was started in 2005 by the NYC Department of Health and Mental Hygiene (DOHMH). In partnership with local community groups, DOHMH District Public Health Offices (DPHOs) distribute $2 coupons — or “Health Bucks” — redeemable for the purchase of locally grown fresh fruits and vegetables at farmers’ markets in the South Bronx, North and Central Brooklyn, and East and Central Harlem (DPHO areas). At farmers’ markets accepting SNAP benefits via the EBT system, consumers have an added incentive. For every $5 in EBT purchases, an additional $2 Health Bucks coupon is provided, to be spent then or at a later time. Local community-based organizations (CBOs) in DPHO areas also distribute coupons directly. We found that Health Bucks participation is associated with increased fruit and vegetable access and purchases in targeted low-income populations (19). Our process evaluation findings cover 3 broad implementation components: 1) coupon distribution and redemption; 2) oversight, management, and evaluation; and 3) manager and farmer/vendor experiences.

## Methods

The purpose of the process evaluation was to identify facilitators and barriers to Health Bucks program implementation. The process evaluation documented program evaluation and early lessons learned and tracked ongoing implementation.

The Health Bucks evaluation design was guided by the CDC Framework for Program Evaluation in Public Health (19). The 2-year study began in September 2009; on-site data collection occurred during active farmers’ market seasons (May–August) in 2009 and 2010. Abt and DOHMH institutional review boards approved all data collection and consent procedures and materials for the evaluation.

The process evaluation collected qualitative and quantitative data from 86 farmers’ markets throughout NYC. During the 2009 farmers’ market season, we conducted formative visits to 1 Health Bucks–participating market in each of the 3 DPHO areas, where we interviewed market staff members about the overall evaluation design. In collaboration with DOHMH staff members, we chose specific farmers’ markets to represent the widest possible array of characteristics, including size, target consumer base, on-site nutrition education activities, and neighborhood (Table 1). During the 2010 farmers’ market season, we conducted evaluative visits to collect data on-site in 86 markets, including the 3 visited in 2009. Details of market sampling are available from the corresponding author; our final sample included 47 markets in DPHO areas, all but one of which participated in Health Bucks, and a stratified random sample of 39 markets outside DPHO areas that did not participate. We excluded 20 markets that opted out of study participation and 3 markets that closed before scheduled data collection. Our final sample included 72% of all farmers’ markets operating in NYC. Our data collection efforts focused on 3 target respondent populations: program administrators, farmers’ market managers, and farmer/vendors.

**Table 1:** Data Collection Activities and Sample Characteristics for Health Bucks Process Evaluation, New York City, 2009–2010

Data Collection Activity/Respondent Group	No. of Respondents	Sampling Strategy and Respondent Characteristics
**Formative data collection (2009 farmers’ market season)**		
**Program administrators**		
Interviews	4	Health Bucks program coordinator
		2 District Public Health Office (DPHO) coordinators
		Implementation contractor
Document review	NA	Administrative reports on issuance and redemption; sample program brochures and materials
**Farmers’ market managers**		
On-site interviews	3	Anonymous respondents at 3 farmers’ markets, 1 per DPHO area. Specific farmers’ markets were chosen in collaboration with NYC DOHMH staff to represent the widest possible array of characteristics, including size, target consumer base, on-site nutrition education activities, and neighborhood.
**Farmers’ market farmer/vendors**		
On-site interviews	4	Anonymous respondents at 3 farmers’ markets, 1 per DPHO area. Specific farmers’ markets were chosen in collaboration with NYC DOHMH staff to represent the widest possible array of characteristics, including size, target consumer base, on-site nutrition education activities, and neighborhood.
**Evaluative data collection (2010 farmers’ market season)**		
**Farmers’ market managers**		
Self-administered surveys	81	Surveys distributed to managers at 86 markets; response rate, 81/86 (94%) Market locations Brooklyn DPHO, n = 10 Harlem DPHO, n = 5 Non-DPHO neighborhoods, n = 59 Market size Average number of vendors: 12 (weekends), 8 (weekdays) Average number of customers: 1,503 (weekends), 1,339 (weekdays) Payment types accepted Cash, 100% Debit/credit card, 49% SNAP benefits/EBT, 77% WIC/senior FMNP coupons, 91% WIC vouchers, 56% Health Bucks, 59% Other forms of payment, 9%
**Farmers’ market farmer/vendors**		
Self-administered surveys	141	Of 282 farmer/vendor surveys distributed in these markets, 192 were completed (68% response rate), representing 141 unique farmer/vendors.
		Percentage who own/operate farm producing goods sold at their stall: 43%
		Percentage selling produce at multiple markets: 79%
		Average number of markets where farmer/vendor sells produce: 3.5
		Percentage selling at a farmers’ market for the first time: 21%
Structured telephone interviews	6	Participants who operated booths/stalls at farmers’ markets accepting Health Bucks were identified by 3 farmers’ market coordinators.
		Average no. of years selling at markets: 14 (range, 6–27)
		Average no. of NYC markets where farmers have sold products: 10.5 (range, 3–22)
		Average approximate percentage of sales from farmers’ markets: 83% (range, 70%–100%)
		No. of respondents selling produce at markets in more than 1 DPHO area: 3

Abbreviations: NA, not applicable; NYC, New York City; DOHMH, Department of Health and Mental Hygiene; SNAP, the Supplemental Nutrition Assistance Program; EBT, electronic benefit transfer; WIC, Special Supplemental Nutrition Program for Women, Infants, and Children; FMNP, Farmers’ Market Nutrition Program.

We interviewed 4 program administrators: the Health Bucks program coordinator at DOHMH, program administrators from 2 of the 3 targeted DPHOs, and a representative of the Health Bucks implementation contractor, the Farmers’ Market Federation of New York (FMFNY). Program administrators provided specific information on program history, current logistics, and operations. Each participated in key informant interviews and provided administrative data and program documents for a document review.

Farmers’ market managers are integrally involved in implementation and day-to-day Health Bucks program operations. They are responsible for program administration and oversight at the market level and often directly distribute coupons to SNAP participants at the market. In some cases, managers serve a dual role as market owner/operator and can provide additional insight into a market’s decision about participating in Health Bucks or accepting SNAP benefits. Data were collected from market managers during 3 key informant interviews on site visits during the 2009 farmers’ market season and via a self-administered written survey distributed to managers in 86 selected farmers’ markets during the 2010 market season, completed by 81 managers (94% response rate).

Farmers’ market vendors operate booths or stalls at each market. Some markets have only 1 vendor, but most have multiple vendors. In some cases, vendors are also farmers who grow or produce items for sale; in others, they are employees hired by the farmer to staff booths. Like market managers, vendors can provide insight into day-to-day program operations, including coupon redemption and reimbursement. Those vendors who are also farmers provided information on factors influencing the decision to sell (or not) at markets in underserved, low-income neighborhoods. Throughout the remainder of this article, this broad group of respondents is referred to as “farmer/vendors” to indicate that the target respondent may fill one or both of these roles.

We conducted key informant interviews with 4 farmer/vendors at 3 participating Health Bucks markets during 2009 site visits to inform development and fielding of a self-administered written survey that was later distributed to all farmer/vendors at the 86 markets where market manager surveys were distributed during the 2010 market season. Of 282 farmer/vendor surveys distributed in these markets, 192 were completed (68% response rate). However, 51 of these surveys were completed by farmer/vendors who had already received and completed a survey at another market, so our final analytic sample included 141 unique farmer/vendors. Although farmer/vendors were instructed to complete the survey only once, because each farmers’ market vendor was identified by a unique number, we were able to identify respondents who inadvertently completed the survey multiple times. However, we could not identify farmer/vendors who did not return a completed survey; therefore, we could not identify the total universe of farmer/vendors operating in study markets.

We also conducted 6 key informant telephone interviews with farmer/vendors after the 2010 season to learn more about their perspectives on the program. Three market coordinators identified participants whose farmers’ market booths accepted Health Bucks.

## Results

**Figure 1:**
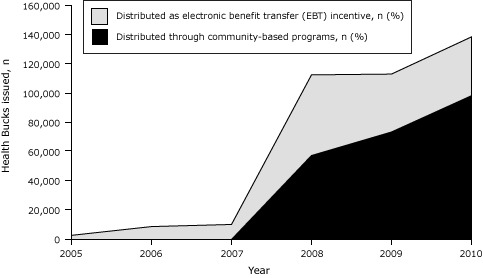
The number of Health Bucks coupons issued as Supplemental Nutrition Assistance Program (SNAP) electronic benefit transfer (EBT) incentives and through community-based organizations, New York City, 2005 through 2010. **Table:** 

Distribution of Health Bucks has increased greatly since program inception, from 3,000 coupons in 2005 to 138,930 coupons in 2010, a 46-fold increase (Figure 1). Because CBOs were the only mechanism for distributing Health Bucks from 2005 through 2007, substantially fewer coupons were distributed in those years compared with 2008 through 2010, when the EBT incentive component of the program was introduced. Since that time, the proportion of coupons distributed as EBT incentives has risen steadily, from 51% in 2008 to 71% in 2010.

Year	2005	2006	2007	2008	2009	2010
**Total Health Bucks issued**	3,000	9,000	10,449	112,919	113,454	138,930
**Percentage distributed as EBT incentive**	0	0	0	51	65	71
**Percentage distributed through community-based organizations**	100	100	100	49	35	29
**No. distributed as EBT incentive **	0	0	0	57,589	73,745	98,640
**No. distributed through community-based organizations**	3,000	9,000	10,449	55,330	39,709	40,290

Redemption rates are higher for coupons distributed on site as EBT incentives than for those distributed by CBOs, although CBO redemption rates have increased over time. Redemption rates for Health Bucks distributed by CBOs rose from 24% in 2005 to 69% in 2010. In comparison, EBT incentive redemption rates have ranged from approximately 85% to just below 90% from 2008 through 2010. Rising distribution and redemption rates have driven accompanying increases in total, per-farmer, and per-market revenues from Health Bucks from 2005 through 2010 (Table 2).

**Table 2:** Health Bucks Coupons Redemptions, New York City, 2005–2010

Year	Participants		Total Redemptions		Average Redemptions a	
	Farmer/Vendor	Markets	No. of Coupons	Dollars	Per Farmer/Vendor	Per Market
2005	15	5	~700	1,400	94	280
2006	30	29	3,600	7,200	240	248
2007	41	33	5,225	10,450	255	317
2008	63	28	79,607	159,214	2,527	5,686
2009	70	50	84,398	168,796	2,411	3,376
2010	81	60	115,686	231,372	2,856	3,856

a Authors’ calculations from total redemptions and number of participating farmers and markets. Source: Farmers’ Market Federation of New York.

Respondents of all types consistently suggested that Health Bucks should be allocated to markets and CBOs as early in the farmers’ market season as possible, allowing distribution over a longer period of time. One program administrator noted that providing coupons earlier in the season helps to establish healthy habits among consumers; the earlier they are introduced to the market, the longer they will benefit each season.

DOHMH employs several oversight policies and processes to standardize stakeholder roles and responsibilities (ie, DPHO coordinators, CBOs, market operators and managers, and vendors) and to enforce required practices for tracking coupon distribution. All participating market operators attended a preseason meeting and received an additional briefing from DPHO coordinators at the beginning of the season. CBOs also received a standardized information packet from DPHO coordinators at the beginning of the season.

Program monitoring systems consisted mostly of mechanisms to track coupon distribution and redemption. Unique bar codes on each Health Bucks coupon allow tracking of distribution and redemption rates. Informal tracking was based on monthly distribution logs completed by both farmers’ markets and CBOs and returned to DPHO representatives. The logs were completed from July to the end of the season and included the coupon bar code number and date for each time a coupon changes hands (eg, from the farmers’ market owner to the market manager, from the market manager to a farmers’ market customer, or from a CBO to a client). However, once the coupons were in the hands of customers, DOHMH could not track them until they were submitted by the customer as payment to the farmer/vendors at the market and returned by the farmer/vendors to FMFNY for reimbursement. At the end of each season, when all coupons used for purchase had been returned, FMFNY compiled summary information on distribution and redemption for the season.

Centralized tracking occurred only at the organizational level; the bar code was not linked to individual Health Bucks recipients. Once distributed, a coupon could be used by any individual shopper at a participating farmers’ market, regardless of whether that individual was the originally intended recipient of the coupon.

As noted above, because farmers do not necessarily redeem coupons on a regular basis, final data on redemption rates were not available until season’s end. In the absence of real-time data on redemptions, DOHMH used distribution logs and prior-year redemption rates to estimate how many coupons have been redeemed and make decisions about how many additional Health Bucks to distribute over the course of the season.

Farmer/vendors accepting Health Bucks submitted collected coupons by mail to the FMFNY for cash reimbursement. Farmer/vendors were typically reimbursed within 6 weeks after submitting Health Bucks. Farmer/vendors generally did not find the Health Bucks reimbursement process burdensome; only about a quarter of farmer/vendor survey respondents reported that the process was not easy, although subsequent farmer/vendor telephone interviews highlighted a few areas for improvement. For example, several farmers reported that they paid out-of-pocket to insure the Health Bucks they mailed to FMFNY, adding a cost of $20 to $40 to postage fees. Interviewees also noted that the smooth texture of Health Bucks made them difficult to count, increasing time required to tally coupons.

DOHMH staff accommodated CBOs by providing computer access to complete online program applications and by continuing to accept applications after the official June deadline. Before 2010, lack of resources to purchase and operate wireless EBT terminals was a major barrier to maximum farmers’ market participation. However, during the 2010 season, a USDA grant allowed all DPHO markets to obtain EBT terminals. In addition, mini-grants from the DOHMH supported staff at smaller markets to operate EBT terminals.

In DPHO areas, acceptance of Health Bucks was identified by farmer/vendors as a market characteristic influencing their decision to sell in 68% of cases. In addition, active community outreach/promotion and acceptance of Food Stamps/EBT benefits were identified as attractive characteristics in DPHO areas.

Farmer/vendors identified 4 specific market characteristics as increasing their propensity to sell at a market; more than half of farmer/vendors identified all 4 characteristics:

- Conducts cooking demonstrations or other nutrition education activities (71%)
- Engages in active outreach or promotion in community (66%)
- Operates on weekends (64%)
- Accepts Food Stamps/EBT benefits (57%)

Only 1 characteristic was selected by at least half of farmer/vendors as a deterrent to selling or operating at a market. Fifty percent of farmer/vendor respondents indicated that “High fees to sell at market” decreased their likelihood of selling at a market.

Farmers’ market managers used multiple methods to promote EBT/SNAP. Nearly all reported that they distributed flyers, brochures, or other promotional handouts (97%) and displayed posters at the farmers’ market (96%). Many engaged in community outreach (88%) or partnered with local CBOs (78%). More than half (52%) used newspaper ads or articles, 36% advertised online, 18% sent direct mailings, and 17% advertised on subways and buses.

Farmers’ market managers generally reported favorable attitudes toward SNAP/EBT. Because their markets accepted EBT, 58% agreed that “more vendors want to operate stands or stalls in this market”; 70% agreed that “new customers shop at this market more often”; and 95% agreed that “more repeat customers come to this market.”

Farmers’ market managers also reported the following attitudes because their markets accepted Health Bucks: 55% agreed that “more vendors want to operate stands or stalls in this market”; 100% agreed that “new customers shop at this market more often”; and 95% agreed that “more repeat customers come to this market.” Lastly, 59% of market managers strongly or somewhat disagreed that because their markets accepted Health Bucks “market traffic moves less smoothly.”

**Figure 2:**
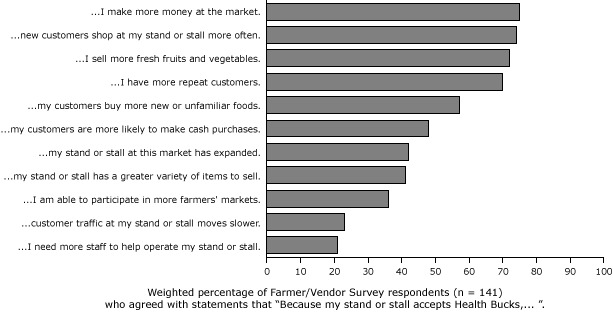
Weighted percentage of farmer/vendor survey respondents (n = 141) who agreed with statements that “Because my stand or stall accepts Health Bucks, . . . ”. **Table:** 

Health Bucks were popular among market farmer/vendors. Most farmer/vendors somewhat or strongly agreed that because they accepted Health Bucks, they made more money at the market (75%), new customers shopped more often at their stand or stall (74%), they sold more fresh fruits and vegetables (72%), they had more repeat customers (70%), and their customers bought more new or unfamiliar foods (57%) (Figure 2). 

Percentage that agree: Because my stand or stall accepts Health Bucks . . .	Agree
I need more staff to help operate my stand or stall	21%
Customer traffic at my stands or stall moves slower	23%
I am able to participate in more farmers’ markets	36%
My stand or stall has a greater variety of items to sell	41%
My stand or stall at this market has expanded	42%
My customers are more likely to make cash purchases	48%
My customers buy more new or unfamiliar foods	57%
I have more repeat customers	70%
I sell more fresh fruits and vegetables	72%
New customers shop at my stand or stall more often	74%
I make more money at the market	75%

## Discussion

Administrators, managers, and farmer/vendors view Health Bucks as a positive program model. Health Bucks distributed as EBT incentives had higher redemption rates and ensured targeting SNAP participants. Distribution through CBOs may have increased awareness of Health Bucks and farmers’ markets among those less familiar with Health Bucks or who did not frequent farmers’ markets regularly. Furthermore, CBO distribution was able to be directly tied to nutrition education and promotion activities. Another implementation facilitator is early distribution. Health Bucks are good throughout the farmers’ market season and may be used even after an EBT balance is depleted. This policy provides nutrition assistance during times of the month when other resources are running low.

This evaluation identified some areas for improving implementation. Program staff described monitoring systems as sufficient, but believed that they could be improved to increase consistency and timeliness of tracking. One potential solution would be to require farmer/vendors to send coupons to FMFNY for redemption on a more regular and frequent basis, though this step would be more burdensome and potentially difficult to enforce. Alternatively, setting up an electronic funds transfer process to replace physical coupons with automated deposits of incentives into EBT accounts would facilitate real-time tracking. In western Massachusetts, the USDA Healthy Incentives Pilot demonstration (currently under way) uses a fully electronic system for depositing incentive payments directly into EBT accounts at the time of purchase, which, if successful, could serve as a potential model (20). This change would facilitate tracking at the individual participant level and ensure that only SNAP participants receive the incentive, reducing potential for fraud. However, it is unclear how this system could accommodate individuals not participating in SNAP who received Health Bucks through CBOs.

Health Bucks tackles health disparities and cost barriers. While in their infancy, farmers’ market incentive programs may be one avenue for increasing access to and affordability of fruits and vegetables among low-income persons. We are not aware of any published evaluations of farmers’ market incentive programs, despite their increased popularity nationwide (21–24). Further research is needed to assess the potential effects of these programs on access and health outcomes. These process evaluation results are intended to facilitate implementation of similar efforts nationwide as part of broader obesity prevention initiatives. Wider implementation, combined with additional information on consumption outcomes, can clarify the effectiveness of farmers’ market incentive programs in reducing disparities in access to and consumption of fresh fruits and vegetables, and improving health outcomes.
